# Validation of noninvasive continuous arterial pressure measurement by ClearSight System™ during induction of anesthesia for cardiovascular surgery

**DOI:** 10.1186/s12871-020-01091-x

**Published:** 2020-07-20

**Authors:** Tadashi Tanioku, Akari Yoshida, Yuichi Aratani, Keisuke Fujii, Tomoyuki Kawamata

**Affiliations:** Department of Anesthesiology, Wakayama Medical University School of Medicine, Kimiidera 811-1, Wakayama, 641-8509 Japan

**Keywords:** Cardiovascular surgery, Non-invasive arterial pressure, Monitoring

## Abstract

**Background:**

Since blood pressure tends to be unstable during induction of anesthesia in patients undergoing cardiovascular surgery, an artery catheter is often inserted before induction to continuously monitor arterial pressure during induction of anesthesia. ClearSight System™ enables noninvasive continuous measurement of beat-to-beat arterial pressure via a single finger cuff without pain using photoplethysmographic technology. If ClearSight System™ can replace intra-arterial pressure measurement, blood pressure could be easily and noninvasively assessed. However, the validity of ClearSight System™ during induction of anesthesia in patients undergoing cardiovascular surgery has not been evaluated. The aim of this study was to compare blood pressure measured by ClearSight System™ with intra-arterial pressure during induction of anesthesia for cardiovascular surgery.

**Methods:**

This study was registered retrospectively. Data during induction of anesthesia for elective cardiovascular surgery were obtained for patients in whom noninvasive arterial pressure was measured by ClearSight System™ (APcs) and invasive radial arterial pressure (APrad) was measured simultaneously. According to the widely used criteria formulated by international standards from the Association for the Advancement of Medical Instrumentation, the acceptable bias and precision for arterial pressure measurements were fixed at < 5 mmHg and 8 mmHg, respectively.

**Results:**

Data for 18 patients were analyzed. For 3068 analyzed paired measurements, values of APcs vs APrad bias (precision) were 13.2 (17.5), − 9.1 (7.3) and − 3.9 (7.8) mmHg for systolic, diastolic, and mean arterial pressures, respectively.

**Conclusions:**

Mean arterial pressure measured by ClearSight System™ could be considered as an alternative for mean radial arterial pressure during induction of anesthesia for elective cardiovascular surgery.

## Background

Since blood pressure tends to be unstable during induction of anesthesia in patients undergoing cardiovascular surgery, an artery catheter is often inserted before induction to continuously monitor arterial pressure during induction of anesthesia. The success rate of the first attempt at arterial cannulation using palpation has been reported to be less than 50% and sometimes cannulation still fails despite the use of ultrasound [1]. Therefore, arterial cannulation in an awake condition may cause suffering for patients.

ClearSight System™ (previously named ccNexfin system™, Edwards Lifesciences Corp, Irvine CA, USA) enables noninvasive continuous measurement of beat-to-beat arterial pressure via a single finger cuff without pain using photoplethysmographic technology. If ClearSight System™ can replace intra-arterial pressure measurement, blood pressure could be continuously, easily, and noninvasively assessed. Previous studies have shown that this device is reliable in pregnant women [2], children [3], and patients undergoing upper abdominal surgery [4]. On the other hand, it has been reported that it is not reliable in critically ill patients [5] and patients undergoing neurosurgery in a sitting position [6]. Accordingly, the validity of ClearSight System™ may depend on the clinical situation including the type of surgery or the patient’s condition. However, the validity of ClearSight System™ during induction of anesthesia in patients undergoing cardiovascular surgery has not been evaluated. The aim of this study was to compare blood pressure measured by ClearSight System™ with intra-arterial pressure during induction of anesthesia for cardiovascular surgery.

## Methods

### Study design and setting

This retrospective observational study was approved by the medical ethics committee of Wakayama Medical University prior to its initiation (reference number 1919). The study was conducted at Wakayama Medical University Hospital.

### Data collection

In this retrospective analysis, data were collected from all patients in whom noninvasive arterial pressure was measured by ClearSight System™ (APcs) and invasive radial arterial pressure (APrad) was measured simultaneously during induction of anesthesia for elective cardiovascular surgery between November 2016 and November 2017 at Wakayama Medical University Hospital. The use of ClearSight System™ depended on the anesthesiologists in charge. Paired values of systolic, diastolic, and mean arterial pressures obtained by both methods were recorded at the rate of 1 sample every 3 s in the institution’s Anesthesia Information Management System (PrimeGAIA™, Nihon Kohden Co, Tokyo, Japan). Data from 5 min before tracheal intubation to 5 min after tracheal intubation based on anesthetic records were analyzed.

In our hospital, the induction of cardiovascular anesthesia has been standardized. Two anesthesiologists are generally involved in one case: one anesthesiologist for managing the anesthesia, and other for recording. Before induction of anesthesia, an catheter is inserted into right radial artery in the most of patients to continuously measure blood pressure. Then anesthesia is induced by target-controlled infusion of propofol (1.5–3.0 μg/ml), remifentanil (0.1–0.3 μg/kg/min), and rocuronium (0.6–1.0 mg/kg). The doses of anesthetics depend on the decision of the anesthesiologist in charge. When blood pressure decreases during the induction, ephedrine (4 mg or 8 mg) or phenylephrine (0.1 mg or 0.2 mg) is intravenously administered according to the decision of the anesthesiologist in charge.

### Statistical analysis

There is no established knowledge of how many patients should be included and how many measurements should be analyzed for each when performing a repeated measurement. In most of the studies using Bland-Altman analysis, the sample size was not examined. In this study, we collected over 3000 pairs of data based on similar previous studies in which radial arterial pressure was compared with blood pressure measured by ClearSight System™ [7, 8].

Data considered to be artifacts were excluded based on ClearSight auto-calibration, radial artery artifacts, and ClearSight artifacts. Auto-calibration is performed at least once every 70 heartbeats to keep the finger arteries open and of constant diameter. In addition, auto-calibration is performed when the measurement of blood pressure is temporarily interrupted for two or more beats. When auto-calibration is performed, systolic, diastolic and mean blood pressures become the same values, and the values increase step by step. Therefore, it is possible to discriminate such data as artifacts. Radial artery artifacts, which result from blood sampling and flushing, could be discriminated since systolic and diastolic pressures become the same values. ClearSight artifacts, which occur due to external pressure to the ClearSight cuff, can be recognized as extreme outliers.

Data are expressed as means (SD) or medians (interquartile range) as appropriate. For whole repeated paired measurements from all patients, correlations between APrad and APcs were determined by a linear regression. In addition, Bland-Altman analysis was used to study agreement between APrad and APcs. In this analysis, bias and precision were defined as the mean difference between APrad and APcs and as the SD of bias, respectively. In addition, limits of agreement (LOA) were calculated as bias ±2SD of bias. According to the widely used criteria formulated by international standards from the Association for the Advancement of Medical Instrumentation (AAMI), the acceptable bias and precision for arterial pressure measurements were fixed at < 5 mmHg and 8 mmHg, respectively [9].

For each patient, the SDs of averages of APrad and APcs (“within-subject variability”) were calculated to quantify the ranges of different pressures. The SDs of differences between APrad and APcs (“within-subject precision”) were also calculated to quantify tracking for systolic arterial pressure (SAP), diastolic arterial pressure (DAP), and mean arterial pressure (MAP). In addition, correlations between APrad and APcs were determined by linear regression.

A two-sided *P*-value of 0.05 was considered statistically significant. All analyses were performed using JMP^Ⓡ^ statistical software (version 12.2; SAS Institute, Cary NC, USA).

## Results

Data for 18 patients were obtained in this study. The characteristics of the patients are shown in Table 1. Given the retrospective nature of this study, all perioperative management was at the direction of the attending clinicians. In all patients, a 22-guage catheter was used for monitoring radial arterial pressure. Both APrad and APcs were measured on the right side in all patients, and noninvasive blood pressure measurement by a cuff was performed on the left arm. Although we obtained 3600 pairs of APcs and APrad, 532 pairs among them were excluded. Of the 532 measurements excluded, 297 measurements were excluded due to ClearSight auto-calibration. In addition, 115 measurements were excluded due to radial artery artifacts, and 120 measurements were excluded due to ClearSight artifacts. The percentage of exclusion data in our data (14.7%) was similar to the percentages in previous prospective studies [7, 8]. Thus, a total of 3068 valid pairs of simultaneous APcs and APrad measurements were analyzed. The median number of paired measurements per patient was 170 (170–200). The ranges of APrad measured during the observational period were 53–225 mmHg for SAP, 27–114 mmHg for DAP, and 41–144 mmHg for MAP. Continuous administration of phenylephrine was started from the beginning of anesthetic induction in 9 of the 18 patients.

**Table 1:** Baseline characteristics of the subjects

Age (yr)	70 (64–79)
Sex (M/F)	11/7
BMI (kg・m^-2^)	24 (21.9–24.9)
Type of surgery (%)	
Coronary artery bypass grafting	11
Aortic valve replacement for aortic valve stenosis	7
Aortic valve replacement for aortic regurgitation	2
Thoracic ascending aortic graft replacement	1
Comorbidities (%)	
Hypertension	18
Diabetes mellitus	9
Renal insufficiency	10
Dialysis	2

*N* = 18. Values are medians (interquartile range) or numbers

*BMI* Body mass index

**Fig. 1:**
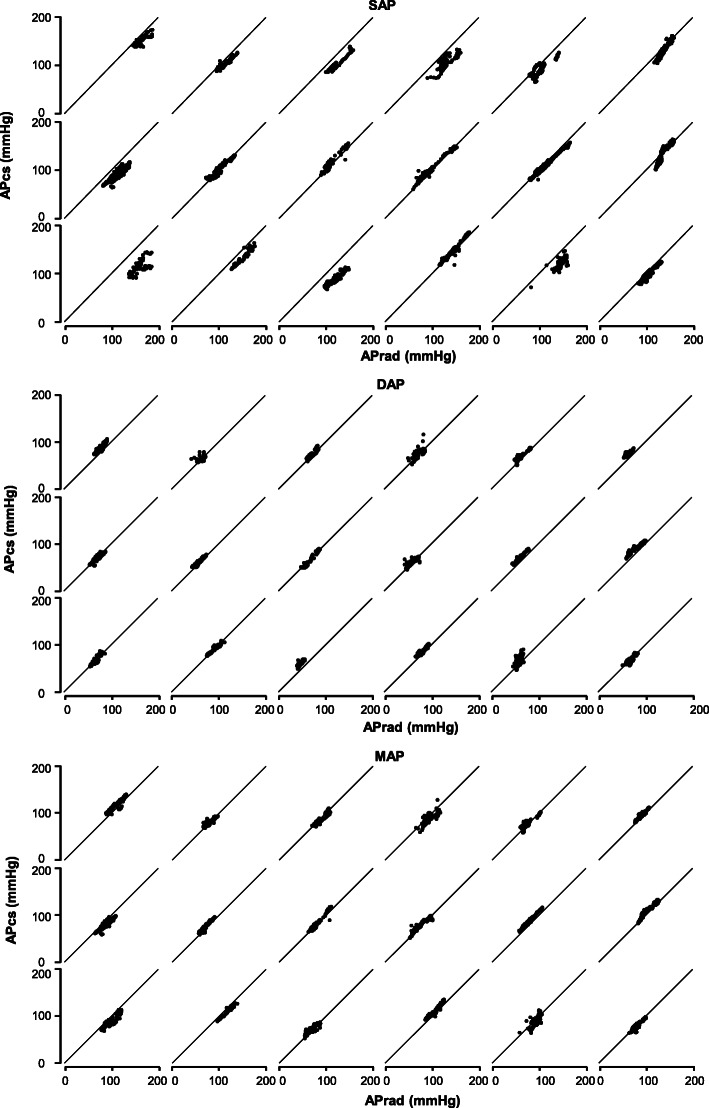
Individual scatterplots of (**a**) invasive and noninvasive systolic arterial pressure, (**b**) invasive and noninvasive diastolic arterial pressure, and (**c**) invasive and noninvasive mean arterial pressure. SAP, systolic arterial pressure; DAP, diastolic arterial pressure; MAP, mean arterial pressure; APrad, invasive radial arterial pressure; APcs, noninvasive arterial pressure measured by ClearSight System™

**Fig. 2:**
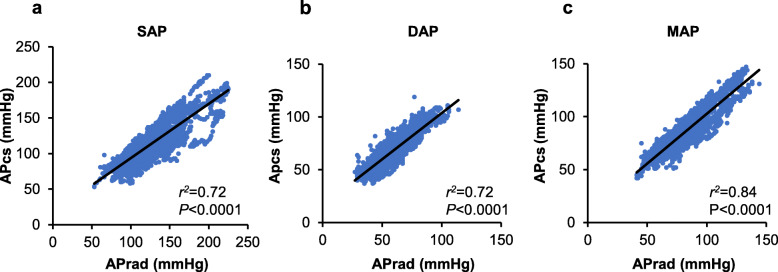
Relationships between absolute values of (**a**) invasive and noninvasive systolic arterial pressure (3084 paired data points), (**b**) invasive and noninvasive diastolic arterial pressure (3084 paired data points), and (**c**) invasive and noninvasive mean arterial pressure (3084 paired data points). r2, coefficient of determination; SAP, systolic arterial pressure; DAP, diastolic arterial pressure; MAP, mean arterial pressure; APrad, invasive radial arterial pressure; APcs, noninvasive arterial pressure measured by ClearSight System™

**Fig. 3:**
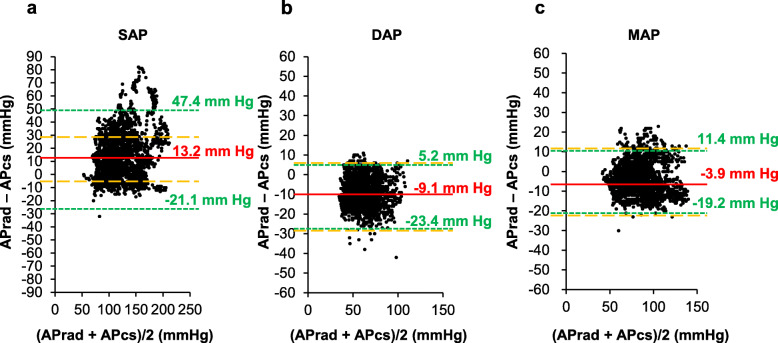
Bland-Altman graphical representation of agreement for individual values of (**a**) systolic arterial pressure between invasive and noninvasive measurements, (**b**) diastolic arterial pressure between invasive and noninvasive measurements, and (**c**) mean arterial pressure between invasive and noninvasive measurements. The red continuous line represents the bias and dotted green lines represent the upper and lower LOA, respectively. Dashed orange lines represent LOA recommended by AAMI for validation of NIAP devices. SAP, systolic arterial pressure; DAP, diastolic arterial pressure; MAP, mean arterial pressure; APrad, invasive radial arterial pressure; APcs, noninvasive arterial pressure measured by ClearSight System™

Figure 1 shows individual scatter plots for SAP, DAP, and MAP. Correlation coefficients, within-subject variability, and within-subject precision are summarized in Table 2. Mean differences of pressure in paired data were 13.1 ± 15.5, − 8.5 ± 6.1, and − 3.4 ± 6.2 mmHg for SAP, DAP, and MAP, respectively. Figure 2 shows the correlations between APcs and APrad. APcs for SAP, DAP and MAP were significantly correlated with APrad. The correlation coefficients between APcs and APrad for SAP, DAP, and MAP were 0.85, 0.85 and 0.92, respectively. The results of Bland-Altman analysis between APcs and APrad are shown in Fig. 3. Bias and precision were 13.2 and 17.5 mmHg in SAP, − 9.1 and 7.3 mmHg in DAP, and − 3.9 mmHg 7.8 mmHg in MAP. Upper and lower LOAs were 47.4 and − 21.1 mmHg in SAP, 5.2 and − 23.4 mmHg in DAP, and 11.4 and − 19.2 mmHg in MAP. Accordingly, only MAP fulfilled the criteria of AAMI.

**Table 2:** Within-subject data averaged over the group

	r, Median (25-75%)	Within-subject Variability	Within-subject Precision
SAP (mmHg)	0.95 (0.89–0.96)	17.3 (4.7)	7.0 (2.6)
DAP (mmHg)	0.91 (0.86–0.95)	8.6 (2.3)	4.1 (1.4)
MAP (mmHg)	0.95 (0.91–0.96)	12.1 (2.7)	4.6 (1.4)

Data are presented as medians (25th-75th percentiles) for correlations and as means (SD) for within-subject variability and within-subject precision in 18 subjects. r, coefficient of correlation

*SAP* Systolic arterial pressure, *DAP* Diastolic arterial pressure, *MAP* Mean arterial pressure

## Discussion

In this study, Pearson’s correlation coefficients showed that SAP, DAP and MAP measured by ClearSight System™ were significantly correlated with radial arterial pressure. Bland-Altman analysis showed that ClearSight System™ had acceptable bias and precision in MAP but not in SAP and DAP for radial arterial pressure measurements. Our results suggest that changes in SAP, DAP and MAP measured by ClearSight System™ reflect those in radial arterial pressure and that MAP measured by ClearSight System™ is interchangeable with radial arterial pressure during induction of anesthesia for elective cardiovascular surgery.

Our study showed that MAP measured by ClearSight System™ statistically matched the AAMI criteria. On the other hand, neither SAP nor DAP matched the criteria. A previous study also showed that MAP, but not SAP and DAP, could be considered as an alternative for radial artery blood pressure during carotid endarterectomy, based on AAMI criteria [8]. In general, the arterial pressure waveform changes gradually from the brachial artery to the finger arteries [10]. Accordingly, SAP at a distal site to the heart is higher than that at a proximal site, while DAP at a distal site is lower than that at a proximal site. ClearSight System™ reconstructs brachial artery pressure from finger artery pressure for calculating blood pressure [10]. Therefore, there might be a significant difference between SAP/DAP measured by ClearSight System™ and radial artery pressure. On the other hand, as blood flows from the aorta to the radial artery, mean pressure decreases only slightly because there is little resistance to flow in the major conducting arteries [11]. In our study, the mean difference in MAP between APcs and APrad was − 3.4 ± 6.2 mmHg, which was small compared to the differences in SAP and DAP (13.1 ± 15.5 and − 8.5 ± 6.1 mmHg, respectively).

The aim of managing hemodynamics is to maintain adequate organ perfusion. MAP is widely used as an index for optimal blood pressure, and it reflects driving pressure at the organ level [12]. MAP is the value that has most often been used for assessing autoregulation of renal blood flow [13] and cerebral blood flow [14]. Measurement of MAP by ClearSight System™ would be useful for maintaining organ perfusion during induction of anesthesia for cardiovascular surgery. During induction of anesthesia for patients with coronary artery disease, maintain of DAP is also important. Our results showed that diastolic pressure measured by ClearSight System™ is not interchangeable with radial diastolic pressure but correlates well with it. Therefore, we can pay attention to coronary perfusion by assessing the change in diastolic pressure but not absolute values measured by ClearSight System™.

The limitation of this study would result from a retrospective nature. The quality of data in a prospective study are generally higher than that in a retrospective study. When APcs is compared to APrad, the advantage of a prospective study is that study conditions including patient’s bias, data collection period, and exclusion of data influenced by artifacts can be controlled. In this study, we decided to analyzed preserved data for the following reasons. First, when we reviewed our preserved data from 18 patients before data analysis, the characteristics of patients were similar to those in previous prospective studies. The exclusion criteria in previous studies included peripheral arterial disease, preoperative atrial fibrillation, and obesity (> BMI 30) [4, 7, 15, 16], and such patients were also not included in our study. Second, when data are collected from the anesthetic record, the time to intubate may not be accurate. In our hospital, two anesthesiologists are generally involved in one case: one anesthesiologist for managing the anesthesia, and other for recording. Therefore, we considered the time of events and data collection period (from 5 min before intubation to 5 min after intubation) would be accurate. Third, in a prospective study, data are excluded due to auto-calibration of APcs, unreliable radial artery wave, flushing arterial line, or APcs artifacts. Among them, auto-calibration of APcs, flushing an arterial line and APcs artifacts can be retrospectively discriminated. Accordingly, the percentage of exclusion data in our data (14.7%) was similar to the percentages in previous prospective studies [7, 8]. In addition, within-subject variability and within-subject precision in our data (Table 2) were similar to those in the previous prospective studies [8, 17], and we therefore considered that the quality of our data is adequate for analysis. For the above reasons, we considered that analysis of preserved data would be less inferior to analysis of prospective data when APcs is compared to APrad. However, a prospective study will be needed to obtain a more precise evaluation of ClearSight System™.

## Conclusions

MAP measured by ClearSight System™ could be considered as an alternative for mean radial arterial pressure during induction of anesthesia for elective cardiovascular surgery. SAP and DAP may be useful for inferring changes in systolic and diastolic radial arterial pressures.

## Data Availability

The datasets used and/or analyzed during the current study are available from the corresponding author on reasonable request.

## Abbreviations

APcs: Noninvasive arterial pressure measured by ClearSight System™
APrad: Invasive radial arterial pressure
LOA: Limits of agreement
SAP: Systolic arterial pressure
DAP: Diastolic arterial pressure
MAP: Mean arterial pressure
AAMI: Association for the Advancement of Medical Instrumentation
